# Calcium at the crossroads of the immunometabolic signaling and comorbidity in schizophrenia

**DOI:** 10.3389/fnsyn.2026.1882307

**Published:** 2026-06-15

**Authors:** Milica M. Borovcanin, Monojit Debnath, Slavica Minic Janicijevic, Ivica Petrovic, Nemanja Muric, Vladimir Janjic

**Affiliations:** 1 Department of Psychiatry, Faculty of Medical Sciences, University of Kragujevac, Kragujevac, Serbia; 2 Psychiatric Clinic, University Clinical Centre Kragujevac, Kragujevac, Serbia; 3 Department of Human Genetics, National Institute of Mental Health and Neurosciences (NIMHANS), Bangalore, India; 4 Special Hospital for Psychiatric Diseases “Kovin”, Kovin, Serbia; 5 Department of Pathophysiology, Faculty of Medical Sciences, University of Kragujevac, Kragujevac, Serbia; 6 Department of Communication Skills, Ethics, and Psychology, Faculty of Medical Sciences, University of Kragujevac, Kragujevac, Serbia

**Keywords:** calcium, immune response, metabolism, schizophrenia, sST2

## 1 Introduction

Calcium (Ca^2+^) channels enable depolarization of the cell surface membrane, allowing Ca^2+^ entry that triggers various secretory functions, contraction, neurotransmission and gene expression, among others. Antipsychotics are known to affect Ca^2+^ signaling and have well-documented metabolic side effects. However, their actions are not the sole cause of Ca^2+^ disturbances in schizophrenia (SCZ). Additionally, both Ca^2+^ and soluble Suppressor of Tumorigenicity-2 (sST2) act as important regulators of immune-metabolic processes. We previously reported a statistically significant positive correlation between serum sST2 concentrations and Ca^2+^ levels, indicating a significant link between Ca^2+^ and the immune-metabolic axis. Increased intracellular Ca^2+^ signaling has been suggested to precede dysregulation of the dopamine system, airway smooth muscle hypercontractility and decreased insulin release. Since sST2 also has established roles in these somatic states and functions as an alarmin in SCZ, it may be a key component in Ca^2+^-regulated immunometabolic signaling and the comorbidity of SCZ.

## 2 Calcium as a trigger *vis-à-vis* a sequel of schizophrenia

**Figure 1:**
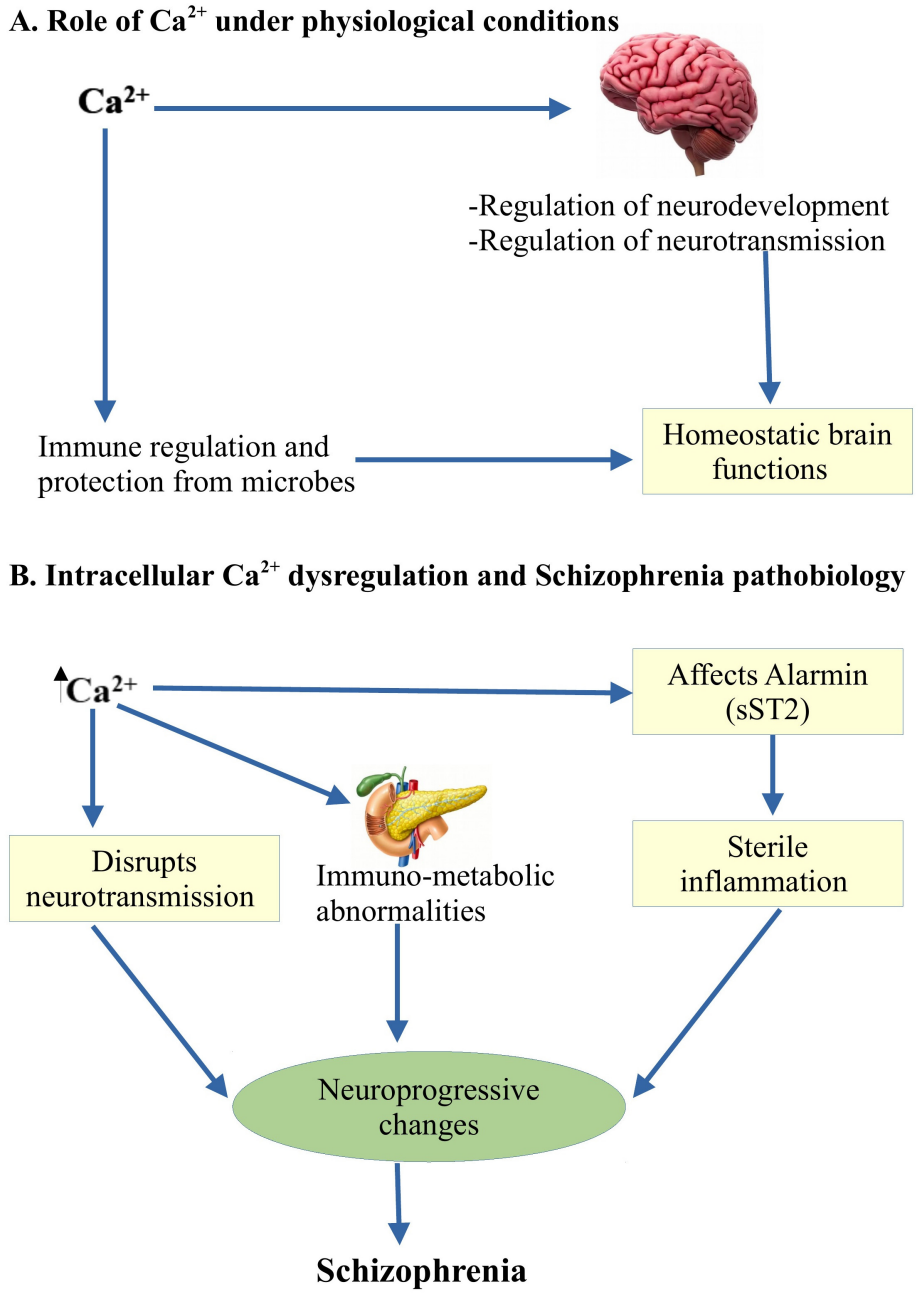
Role of Ca^2+^ under physiological conditions and dysregulation in schizophrenia. **(A)** The role of Ca^2+^ in physiological conditions involves the regulation of neurodevelopment, neurotransmission, and immune functions, contributing to brain homeostasis. **(B)** In schizophrenia, Ca^2+^ is involved in disrupted neurotransmission, which also affects the alarmin sST2, leading to sterile inflammation and immuno-metabolic abnormalities. These factors contribute to neuroprogressive changes in schizophrenia. Ca^2+^, calcium; sST2, soluble suppressor of tumorigenicity.

Ca^2+^ channels facilitate depolarization of the cell membrane, allowing entry of Ca^2+^ into the cells. This entry triggers various cellular functions, including gene expression, secretion, contraction in neuronal cells, and plays a key role in neurotransmission (Figure 1A). Notably, mutations and polymorphisms in Ca^2+^ channels have been associated with the onset of various neuropsychiatric diseases, including migraine, cerebellar ataxia, autism, depression, bipolar disorder and SCZ (Nanou and Catterall, 2018).

Hypocalcaemia was consistently observed in patients with SCZ, and it was considered in clinical practice to be a sequela rather than a causal determinant. Interestingly, analysis of a large cohort of patients with SCZ reported an association between hypocalcaemia and use of the atypical antipsychotic risperidone (Milovanovic et al., 2010). Antipsychotics lead to secondary metabolic effects through modulation of the immune system and bone metabolism, that are evident in patients with SCZ (Koricanac et al., 2023). Recent interpretations of the akathisia phenomenon, characterized by subjective inner restlessness and often accompanied by anxiety, discomfort, and objective signs such as rocking, fidgeting, leg shuffling, pacing, or inability to sit still, also indicate the involvement of antipsychotics' Ca^2+^ channel blockade (Thippaiah et al., 2021). Evidence from a Mendelian randomization study suggests a link between Ca^2+^ regulatory hormones and psychiatric phenotypes, indicating that innate differences in Ca^2+^ regulation could influence both disease biology and sensitivity to drug-induced movement disorders (Jiang et al., 2023).

Although Ca^2+^ metabolism has primarily been discussed by clinicians in the context of antipsychotic treatment, current understanding suggests its diverse roles in both physiological and exposome-associated processes in neuropsychiatric conditions (Figure 1). A host of factors, such as unhealthy lifestyle, reduced physical activity, smoking, poor diet, and genetic elements, confer vulnerability to SCZ. It is noteworthy that a substantial number of patients with SCZ are predisposed to the cumulative side effects of medications. Contextually, all these vulnerability factors, as well as antipsychotic-induced side effects, also contribute to systemic changes, especially in immune and metabolic trajectories in SCZ (Dickerson et al., 2006).

## 3 Immuno-regulatory roles of calcium in schizophrenia: new perspectives

Findings from our recent studies provide interesting insights into the immune connections of Ca^2+^ in SCZ. We demonstrated elevated levels of soluble Suppressor of Tumorigenicity-2 (sST2), a member of the IL-1 family, in the exacerbation of psychosis (Borovcanin et al., 2018). When it binds to IL-33, sST2 dampens the effects of IL-33. Soluble ST2 (sST2), as an alarmin, was found to exert beneficial effects by negatively correlating with scores reflecting negative symptoms of SCZ. In a subsequent study of 27 SCZ patients in remission, we observed an association between sST2 and Ca^2+^. There was a statistically significant positive correlation between serum sST2 concentrations and Ca^2+^ levels (*r* = 0.454; *p* = 0.020) (Minić Janićijević, 2024). These findings suggest a connection of Ca^2+^ with a key substrate of sterile inflammation in SCZ. Based on this understanding, we suggest that Ca^2+^ has the potential to emerge as an important regulator of immune-metabolic signaling in SCZ (Figure 1B).

## 4 Calcium as the driver of comorbidity in schizophrenia arising from immunometabolic dysregulation

Ca^2+^ is at the intersection of multiple signaling pathways. The review article by Bergantin (2020) provided critical insights into the functional implications of dysregulated intracellular Ca^2+^ concentration released from the Endoplasmic Reticulum (ER). Such an understanding has been crucial in unfolding the basis of Cyclic Adenosine Monophosphate (cAMP) stimulation in SCZ, asthma, and diabetes. Dysregulation of Ca^2+^ signaling, specifically increased intracellular concentration, may precede dysregulation of the dopamine system, airway smooth muscle hypercontractility and decreased insulin release.

We have identified similarities between the immune responses in asthma, as a type 2 immune response prototype, and SCZ. Patients with SCZ seem to have a more pronounced type 2 immune response (Borovcanin et al., 2012), as indicated by elevated sST2 during exacerbations (Borovcanin et al., 2018), that may be corrected with antipsychotics (Borovcanin et al., 2013). Repeated exacerbations in both diseases, accompanied by inflammation in the microvascular network, could lead to deterioration of joint tissues, fibrosis and gliosis (summarized in Borovcanin, 2020). Importantly, one of the etiological models of asthma proposed Ca^2+^ elevation within the ER as an endogenous inducer of inflammation (Cantero-Recasens et al., 2010). Consistent with our findings, *in vitro* studies observed that ST2 was less expressed in bronchial epithelial cells, and it was explained that stimulation with IL-33 increased intracellular Ca^2+^ in this proinflammatory environment (Kaur et al., 2020).

In neurons and pancreatic β-cells, intracellular Ca^2+^ homeostasis and ER function are important determinants of cellular function. In SCZ, one of the most important molecules linking these processes is the protein Disrupted-In-Schizophrenia 1 (DISC1), which has been identified as a significant susceptibility gene for SCZ (Camargo et al., 2007). The literature indicates that DISC1 is localized to the ER membrane and functionally interacts with components that regulate Ca^2+^ release. Loss of DISC1 function leads to dysregulation of Ca^2+^, with increased release of Ca^2+^ into the cytosol (Park et al., 2015). Based on these findings, DISC1 acts as a fine-tuning regulator of Ca^2+^ release from the ER and of intracellular Ca^2+^ dynamics, while dysfunction of DISC1 leads to significant disruption of intracellular signaling. These disorders are accompanied by disruption of synaptic homeostasis and of dopaminergic and glutamatergic neurotransmission, which are central characteristics of neurotransmission in SCZ (Rittenhouse et al., 2021) (Figure 1B).

Under conditions of chronic β-cell hyperinsulinemia and ER stress, the synthesis of proinsulin and the ER's regulatory role in maintaining intracellular Ca^2+^ homeostasis are disrupted. This is accompanied by increased Ca^2+^ “leakage” from the ER and a resulting chronic rise in intracellular Ca^2+^ (Dobson and Jacobson, 2024). This imbalance may be initialized with enhance insulin secretion, but over time, it leads to depletion of secretory capacity and functional decompensation of β-cells. Chronically elevated cytosolic Ca^2+^ activates calcium-dependent proteases, mitochondrial pathways, and oxidative stress, leading to activation of apoptotic mechanisms and loss of β-cell mass (Zhang et al., 2020) (Figure 1B). Additionally, chronic low-grade inflammation through secreted cytokines further enhances ER stress and destabilizes Ca^2+^ homeostasis (Brozzi et al., 2015). In this context, sST2 should also be considered as a contributing factor to metabolic syndrome. Under physiological conditions and for maintaining metabolic homeostasis, IL-33 activates regulatory T cells (Tregs) and type 2 Innate Lymphoid Cells (ILC2s), thereby promotes anti-inflammatory milieu and adipose thermogenesis. In contrast, in obesity, the “expression-function paradox” is observed (Cui et al., 2025). This means that although IL-33 expression is elevated in obese adipose tissue, effector cell dysfunction impairs its protective attributes, including sST2-mediated neutralization.

## 5 Clinical implications

The above observations may help explain the specific vulnerability to SCZ and the involvement of Ca^2+^ not only in the early programming but also in the orchestration of a unique immunometabolic and mental profile later in life (Pillinger et al., 2019). It should also be considered that cumulative effects could lead to neurodegeneration and neuroprogression in patients with SCZ (Davis et al., 2016). Frequent exacerbations and deterioration observed in SCZ may be linked to disturbances in Ca^2+^/ST2 dynamics. Inflammation and Ca^2+^ homeostasis are among the “hallmarks of aging” pathways used to identify potential frailty biomarkers (Cardoso et al., 2018).

A considerable number of SCZ patients have chronic low-grade inflammation, and it is suggested that they constitute an inflammatory subgroup of SCZ (Hoang et al., 2022). Patients with SCZ exhibited specific immune reactivity, with notable differences observed in those resistant to conventional therapy. The immune markers are increasingly suggested as potential targets of pharmacotherapy in SCZ. IL-33/ST2 signaling pathway is emerging as one of the viable new targets of various neuroinflammatory conditions. The sST2 can be considered as a rational target of pharmacotherapy in SCZ and other neuroinflammatory conditions, as targeting sST2 can attenuate neuroinflammation and promote repair mechanisms in the brain.

In recent years, several novel antipsychotic drugs have demonstrated significant immune-modulatory properties. For example, the Trace Amine-Associated Receptor 1 (TAAR1) agonist was demonstrated to reduce microglia-mediated inflammatory response (Polini et al., 2023). TAAR1 acts as an inhibitory modulator of the dopaminergic system and is involved in inducing neuroinflammation and microglia activation (Tsytsarev et al., 2025). Additionally, it is considered a promising new target for improving β-cell health in type 2 diabetes mellitus (Michael et al., 2019). Notably, TAAR1 impacts the signaling and internal mobilization of Ca^2+^ (Navarro et al., 2006; Ohno et al., 2025). The crosstalk amongst TAAR1, Ca^2+^ and IL-33/ST2 pathway needs further exploration in SCZ. Improvement of the negative and cognitive symptoms with more viable anti-inflammatory or immunomodulatory agents should be the main focus of future pharmacotherapeutic strategies.

These findings need to be incorporated more rapidly into clinical practice for the benefit of patients. A better understanding of Ca^2+^-dependent immune-metabolic signaling may improve the identification of biologically distinct SCZ phenotypes and patients at increased risk of metabolic comorbidity. Future studies should determine whether Ca^2+^/ST2 interactions could serve as clinically relevant biomarkers or therapeutic targets in SCZ.
